# Asymmetric Implant Design for Posterolateral Overhang of the Femoral Component in Total Knee Arthroplasty: A Retrospective Computed Tomography-Based Study

**DOI:** 10.7759/cureus.56862

**Published:** 2024-03-25

**Authors:** Masashi Hirakawa, Masashi Miyazaki, Yu Nagashima, Hiroya Akase, Shogo Matsuda, Nobuhiro Kaku

**Affiliations:** 1 Department of Orthopaedic Surgery, Oita University, Yufu, JPN

**Keywords:** knee surgery, popliteus tendon, overhang, computed tomography, total knee arthroplasty

## Abstract

Introduction

During total knee arthroplasty (TKA), also referred to as total knee replacement (TKR), patients may experience pain in the posterolateral knee. One possible cause is the impingement between the popliteus tendon and the femoral components. The purpose of this study was to analyze the posterolateral overhang of the femoral component using 3D template software.

Methods

Preoperative CT scan images of 50 knees (11 males and 39 females) with osteoarthritis of grade 2 or lower according to the Kellgren-Lawrence classification were analyzed. The mean age of the subjects was 73.8±7.6 years (range 52-84 years). The Athena (Soft Cube Co., Ltd., Osaka, Japan) knee 3D image-matching software was used for the analysis. The positions of the two femoral components (symmetrical and asymmetrical) were simulated. In the coronal plane, the component overhang was measured between the resected lateral part of the posterior femur and its corresponding component size, and the two designs were compared in three zones (proximal, central, and distal).

Results

In the simulated femoral component, the asymmetric design had a significantly lower component overhang than the symmetric design in the proximal zone of the lateral posterior condyle (0.2±1.9 mm vs. 3.5±1.6 mm, p<0.01). In the proximal zone, significant overhang (>3 mm) was observed in 30 knees (60.0%) with the symmetric design, but only three knees (6.0%) had asymmetric designs (p<0.01).

Conclusions

The posterolateral overhang of the lateral posterior condyle occurs when a symmetrical prosthesis is used. The use of an asymmetric implant with a small, rounded proximal portion of the lateral posterior condyle improves this overhang and is expected to decrease problems such as impingement of the popliteus tendon and improve patient satisfaction.

## Introduction

Total knee arthroplasty (TKA) is a highly effective procedure for pain relief and restoration of knee function in patients with end-stage degenerative and rheumatological knee joint diseases [1-5]. The average rate of total knee arthroplasty, including primary and revision, is 175 per 100,000 people, with the rate increasing markedly over time [1]. Nevertheless, it has been reported that 11-19% and 31% of patients with primary TKA and those with revision TKA, respectively, are not satisfied with their outcomes [6,7].

Accurate component sizing and surface design are essential for the success and long-term survival of TKA [4-7]. Currently, available prosthesis designs are developed based on the natural geometric and kinematic properties of the knee. The posterior condyle of the normal knee joint is medially and laterally asymmetric, thereby exhibiting a pivoting motion of the femur in external rotation relative to the tibia. However, the femoral geometry of most prostheses is designed such that the distal and posterior condyles are symmetrical, which can alter the rotation pattern of the knee postoperatively and affect knee joint function [8,9].

Ideally, in TKA, the condyles of the prosthesis should fit the contours of the resected bone, and mismatches between the bone and implant, especially overhangs and oversizing, can worsen clinical outcomes [10,11]. Most prostheses currently on the market are designed such that the bone-implant contact surface is symmetrical at the posterior femoral condyle [9]. However, a high frequency of posterolateral overhang has been observed [10-12]. This mismatch may cause femoropopliteal impingement following TKA [13-15], suggesting that, although the shape of the femoral component is symmetrical, the shape of the resection surface of the femoral posterior condyle is asymmetrical, resulting in a mismatch. However, to the best of our knowledge, few studies have examined the extent to which the overhang at the femoral posterior condyle differs between symmetric and asymmetric implants. We hypothesized that the use of asymmetric implants results in a lesser overhang of the posterior lateral femoral condyle than that of symmetric implants. This study aimed to simulate femoral component placement using 3D preoperative planning software to identify differences in the overhang of the femoral posterolateral condyle between symmetric and asymmetric implants.

## Materials and methods

Study design and patients

The study was conducted at Oita University, Yufu, Japan. The study protocol for this retrospective and noninvasive study was approved by the institutional review board of our hospital (approval No. 1850). Informed consent was obtained in the form of an opt-out, and the need to obtain informed consent from individual patients was waived by the ethics committee of our institution.

Computed tomography (CT) images of 45 patients and 50 knees (11 males and 39 females) with knee osteoarthritis were analyzed using computer software. The mean age of the subjects was 73.8±7.6 years (range 52-84 years), and the Kellgren-Lawrence classification grade (K-L grade) was grade 2 or less with minimal degeneration. Patients with valgus knee, those with a history of fracture, those with previous lower extremity surgery other than arthroscopy, those with a severe deformity of ≥195° in hip-knee-ankle angle (HKA), or those with severe osteoporosis were excluded.

Analysis methods

The CT data for the entire femur length used in the analysis were 1 mm slices to ensure sufficient data quality for 3D reconstruction. Evaluations were performed using the Athena (Soft Cube Co., Ltd., Osaka, Japan) knee 3D image-matching software [16,17]. This software can display bones in 3D space based on CT data. By displaying the 3D shape data of the implant in a 3D space where the bone is displayed, it is possible to simulate the size and placement position of the implant, and it is possible to measure the amount of osteotomy, the angle between the implant, and the functional axis using length and angle measurement functions.

**Figure 1:**
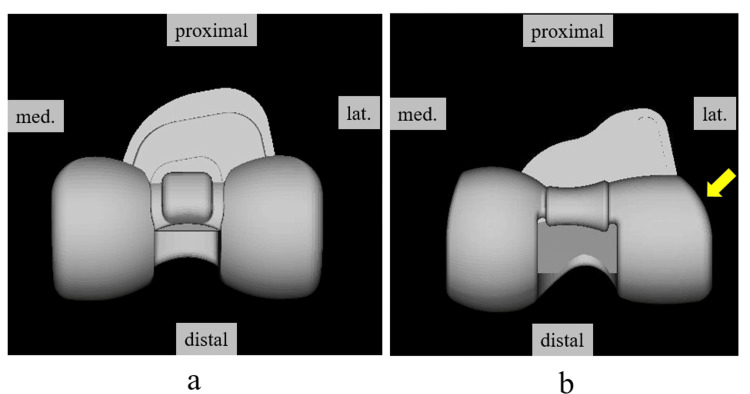
Femoral components used in the simulation a) Symmetric design: Vanguard PS (Biomet Inc., Warsaw, USA); b) Asymmetric design: FINE knee PS (Teijin Nakashima Medical, Okayama, Japan). The lateral condyle is smaller than the medial condyle, and in the femoral component, the posterior lateral shape is round and reduced (arrows).

The simulation was performed using two different component templates: symmetric and asymmetric. The symmetric component was the Vanguard Posterior Stabilized (PS) (Biomet Inc., Warsaw, USA), and the asymmetric component was the FINE knee PS (Teijin Nakashima Medical, Okayama, Japan). The FINE knee has an anatomical shape; the lateral condyle is smaller than the medial condyle, is rounded in shape, and has reduced the shape of the posterolateral corner of the femoral component (Figure 1).

**Figure 2:**
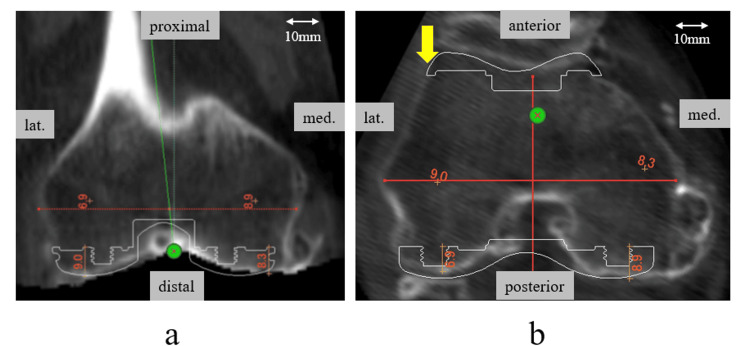
Simulated location of the femoral components a) Coronal plane: The osteotomy line was set perpendicular to the femoral mechanical axis and 9 mm proximal to the distal end; b) Axial plane: The components set the osteotomy line parallel to the s-TEA. The mediolateral (ML) location of the components was determined such that the lateral end of the femoral component was just on the lateral cortex of the femur (arrow). The size of the component was selected so that the osteotomy of the medial posterior condyle was 9 mm ± 1 mm, the thickness of the component, without creating a notch on the anterior surface of the femur.

The anatomical coordinate system of the femur was determined based on several bony landmarks [18]. The midpoint of the surgical transepicondylar axis (s-TEA), which is the line connecting the sulcus of the medial epicondyle to the lateral epicondyle prominence of the femur, was used as the origin for the femoral coordinate system. The femoral mechanical axis was defined as the line from the center of the femoral head to the midpoint of the s-TEA. The components were placed perpendicular to the femoral mechanical axis in the coronal plane and 9 mm proximal to the most distal point of the femoral medial condyle. In the sagittal plane, the components were placed parallel to the anatomical axis of the distal femur (the line connecting the midpoints of the femoral width at 100 mm and 150 mm proximal to the distal femur). In the axial plane, component rotation was parallel to s-TEA. Since the s-TEA could not be evaluated on imaging in some cases, it was defined as the position of 3° of internal rotation from the clinical transepicondylar axis (c-TEA) connecting the most prominent points of the medial and lateral epicondyles of the femur, based on the reports of other authors [19,20]. The size of the component was selected so that the osteotomy of the medial posterior condyle was 9 mm ± 1 mm, the thickness of the component, without creating a notch on the anterior surface of the femur. The mediolateral locations of the components were determined such that the lateral end of the femoral component was located on the lateral cortex of the femur in the axial plane (Figure 2). Additionally, the femoral condylar transverse width and the condylar twist angle (CTA) were also measured. The femoral condylar transverse width was determined in the coronal plane on the 3D template and is defined as the distance between the prominences of the medial and lateral femoral epicondyles. The CTA is the angle formed between the posterior condylar line (PCL), a line running across the tips of the two posterior condyles, and the c-TEA. In this simulation, the rotational placement of the component was parallel to the s-TEA.

**Figure 3:**
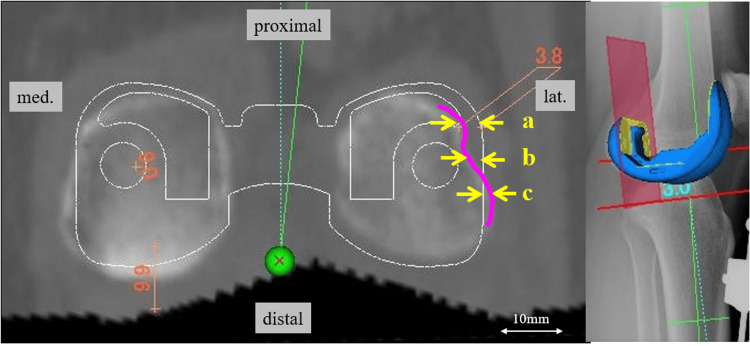
Method for measuring overhang at the posterior femoral condyle The posterior condylar osteotomy plane was drawn in the coronal plane. The amount of component overhang was measured as the difference between the resected medial and lateral parts of the posterior femur and its corresponding components. The overhanging area was equally divided into three zones (proximal, central, and distal). The two-component designs (symmetric and asymmetric) were compared in three zones. Overhangs are indicated by plus and underhangs by minus.

Coronal-sectional images of the posterior condylar osteotomy plane were used for these measurements. The amount of component overhang was measured as the difference between the resected medial and lateral parts of the posterior femur and its corresponding components. The overhanging area was equally divided into three zones (proximal, central, and distal). Each zone was defined as follows: proximal, the most proximal part of the lateral line of the component posterior condyle; central, the middle of the proximal and distal; and distal, the most distal part of the femoral posterior condylar surface. The two-component designs (symmetric and asymmetric) were compared in three zones (Figure 3). Evaluations were performed by two authors who were blinded to the clinical information, and the averages were used.

Statistical analysis

Intra- and inter-observer measurement reliabilities were analyzed using intraclass correlation coefficients (ICC). For descriptive analysis, means and standard deviations (SD) were calculated. Differences in the data were statistically analyzed using the Mann-Whitney U test and the chi-square test. Power analysis was performed to determine the sample size from similar studies. The minimum sample size was determined to be 51 to provide a statistically significant difference with 0.80 power, a 0.05 error margin, and a 0.5 effect size. Statistical significance was set at p<0.05. All statistical analyses were performed with IBM SPSS Statistics for Windows, Version 18 (released 2009; IBM Corp., Armonk, New York, United States).

## Results

Intra-observer and inter-observer variability

All ICC values were >0.87, indicating a very high intra- and interobserver reliability.

Patient demographics and the overhang of the posterior femur

Patient demographics are presented in Table 1. It was estimated that the osteotomy of the lateral posterior condyle was smaller and the overhang was larger in cases with a larger CTA. However, no correlation was found between each parameter (sex, body size, K-L grade, femoral condylar transverse width, and CTA) and the overhang of each posterior condyle.

**Table 1:** Patient demographics used in CT data CTA: condylar twist angle

Characteristics	All Subjects, N＝50
Age (mean ± SD, years)	73.8 ± 7.6
Gender (male)	11
Gender (female)	39
Height (mean ± SD, cm)	155.7 ± 7.8
Weight (mean ± SD, kg)	61.9 ± 10.3
Body-mass index (mean ± SD, kg/m^2^)	27.2 ± 6.5
K-L grade 0	1
K-L grade 1	13
K-L grade 2	36
Femoral condylar transverse width (mean ± SD, mm)	74.8 ± 4.5
CTA (mean ± SD, °)	7.7 ± 1.4

After the simulation of the femoral component, there were no significant differences in the amount of overhang between the symmetric and asymmetric components of the medial posterior condyle (Table 2). In the lateral posterior condyle, there were no differences at the central and distal parts, but there was a significant difference in the amount of overhang between the symmetric and asymmetric components at the proximal part of the lateral posterior condyle (3.46±1.57 mm vs. 0.22±1.85 mm, p<0.001).

**Table 2:** Results of medial and lateral overhang for posterior condyles Data are presented as means, standard deviations, and ranges; ** p<0.001

Femoral component		Symmetric			Asymmetric			
		Mean (mm)	SD	Range	Mean (mm)	SD	Range	p-value
Medial	Proximal	0.38	2.83	-6.9 to 6.5	0.07	2.08	-4.7 to 4.5	0.402
	Center	-0.69	2.75	-7.8 to 4.4	-0.47	1.86	-4.1 to 3.5	0.812
	Distal	-1.23	2.57	-8.7 to 4.8	-0.92	1.78	-4.2 to 2.3	0.553
Lateral	Proximal	3.46	1.57	0.2 to 6.9	0.22	1.85	-4.6 to 4.6	<0.001*
	Center	1.57	1.68	-2.3 to 5.9	1.40	1.68	-3.0 to 4.4	0.759
	Distal	-0.20	1.53	-3.4 to 4.3	-0.01	1.49	-3.2 to 3.6	0.450

Furthermore, we defined a significant overhang as an overhang of 3mm or more based on previous reports [10]. Significant overhang was observed in 30 knees (60.0%) in the symmetric component but only in three knees (6.0%) in the asymmetric component in the proximal part of the lateral posterior condyle. There was a significant difference between the two groups only in the proximal part of the lateral posterior condyle (p<0.001); there were no differences between the two groups in other parameters (Table 3).　

**Table 3:** Number and percentage of significant overhangs (≥3 mm) in the posterior condyle ** p<0.001

Femoral component		Symmetric (n=50)	Asymmetric (n=50)	p-value
Medial	Proximal	9 (18.0%)	2 (4.0%)	0.055
	Center	5 (10.0%)	2 (4.0%)	0.433
	Distal	1 (2.0%)	0 (0%)	1.000
Lateral	Proximal	30 (60.0%)	3 (6.0%)	<0.001**
	Center	10 (20.0%)	11 (22.0%)	0.806
	Distal	1 (2.0%)	2 (4.0%)	1.000

## Discussion

The primary finding of this study is that the shape of the medial and lateral posterior condyles after femoral posterior condylectomy is asymmetric and that the use of symmetric components results in a high percentage of overhang proximal to the lateral posterior condyle, which can be reduced by optimizing the shape proximal to the lateral posterior condyle.

Pain after knee replacement surgery is common and often leaves patients with significant symptoms with no specific explanation [21]. It has been reported that 15-20% of patients remain symptomatic and unsatisfactory after knee replacement surgery [22]. The reasons for this observed dissatisfaction are heterogeneous, with surgery-specific factors including surgical technique and implant design.

Ideally, in TKA, the condyle of the prosthesis should fit the contours of the resected bone. Mismatches between the bone and implant, especially overhangs and oversizing, can worsen clinical outcomes. Mahoney et al. [10] reported that a femoral component overhang of >3 mm in at least one zone was associated with an almost two-fold increased risk of knee pain that was more severe than occasional or mild at two years after surgery (odds ratio: 1.9). Bernard-de Villeneuve et al. [23] examined whether modern morphometric components with more sizes available had a better femoral component fit, bone coverage, and clinical advantages than the femoral components of earlier designs with fewer sizes. The use of a morphometric femoral component design showed slightly improved bone fit and pain scores according to the Knee Society Score (KSS) at midterm follow-up compared to earlier implants of smaller sizes. Furthermore, an overhang of >2 mm was associated with a worse Knee Injury and Osteoarthritis Outcome Score (KOOS). Despite these reports, it is still unclear which part of the overhang is responsible for the pain because it has not been examined.

**Figure 4:**
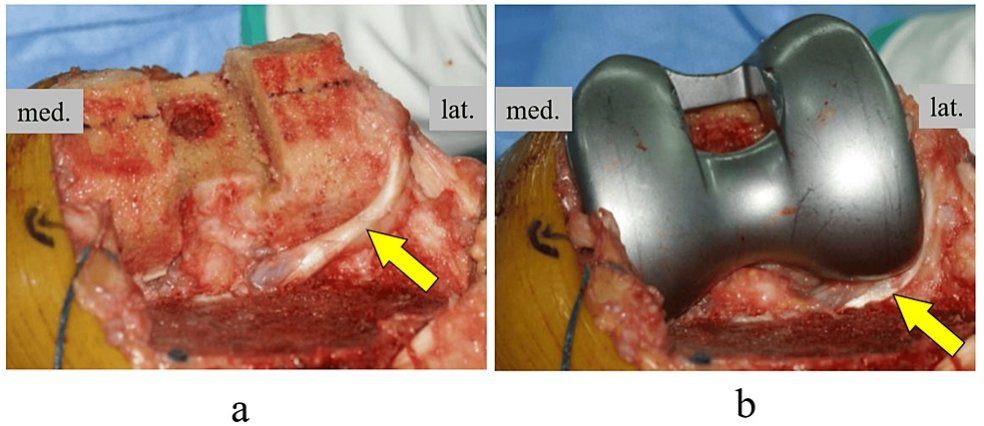
Popliteal tendon after intraoperative osteotomy and component-induced impingement a) Post-femoral osteotomy: The popliteal tendon is partially in contact with the posterior condylar osteotomy site; b) After the femoral component trial placement, the popliteal tendon was compressed and shifted posteriorly by the component.

The posterolateral femoral overhang can worsen clinical outcomes by increasing tension and friction on the ligaments, capsule, and popliteus tendon. The popliteus tendon is considered particularly prone to impingement owing to its intra-articular location and close contact with the lateral femoral condylar margin [24]. Popliteus tendon impingement (PTI) is a soft tissue impingement after TKA (Figure 4), and PTI has been described as secondary to friction with the femoral osteophyte [13,25] or overhang of the prosthesis [14,15], which is successfully treated by surgical release. The presence of localized posterolateral pain has been reported as a diagnostic of PTI. Barnes et al. [25] describe PTI as an intraoperative phenomenon recognized by the presence of palpable and/or audible "snipping" of the tendon, and Bonnin et al. [26] describe it as pain reproduced when palpating the joint from 90° flexion to full extension of the knee joint. Since the patella is moved laterally in the usual TKA procedure, it is difficult to intraoperatively observe whether there is an overhang in the posterior lateral femur, where the popliteus tendon runs. This makes the intraoperative detection of PTI difficult.

The present study suggested that in symmetrical implants, an overhang of the lateral posterior condyle may occur proximally, where intraoperative identification is more difficult, even if it is not observed distally. The popliteal groove is present proximal to the outer osteotomy surface of the posterior femoral condyle and is more concave than the distal bony surface. Since the popliteus tendon fits into this groove during knee flexion, it can be inferred that an overhang proximal to the lateral posterior femoral condyle increases the risk of PTI. This explains the occurrence of posterior lateral overhang after TKA. However, the PTI was improved by an asymmetric component with a rounded and reduced shape of the lateral femoral posterior condyle. Modifying the posterior condyle of the prosthesis to match the irregular shape of the posterolateral resection surface reduces the occurrence of posterior lateral overhang after TKA and also reduces residual knee pain and stiffness. On the other hand, underhanging of the femoral component could leave cancellous bone exposed, which may be a source for postoperative bleeding or maybe an instigating site for osteolysis when wear debris is present [27]. However, the amount of underhang at this site is expected to be much smaller than at the distal femur or tibia, so the clinical problem is not expected to be significant.

In the present study, a 3D computer modeling method using computed tomography (CT) was used to evaluate the posterior femoral condyle in TKA. The advantage of this 3D computer model is that accurate morphological information can be obtained with good reproducibility. However, several limitations of the present study must be considered. First, the present study included only osteoarthritis (OA) knees. However, since most TKA procedures are performed on arthritic knees, OA knee measurements are better suited for designing suitable prostheses. Second, data were obtained only from the Japanese population. Future studies should include samples from other racial groups. Third, the shape of the posterior femoral condyle varies with each implant. Even implants that are considered to have the same symmetry may have different posterior condyle shapes, so the results of this study may not apply to all implants. Fourth, all measurements were made using CT data that did not include cartilage thickness data. Magnetic resonance imaging (MRI) is necessary to accurately assess the articular surface, including the cartilage thickness. However, MRI cannot be used to evaluate the entire length of the femur. The CT data used in the present study included the entire length of the femur, which is advantageous for constructing an accurate TKA-based coordinate system compared to previous studies using MRI data [9]. Fifth, the results of the present study may vary depending on the alignment of the femoral components. For example, if the femoral component is placed externally in the axial plane, the amount of osteotomy of the lateral posterior femoral condyle is expected to be even smaller, and the overhang is expected to be larger. In addition, in recent years, there have been various opinions on proper alignment in TKA, and the results would be different if the placement alignment of the femoral component were completely different from that in this study, as in kinematic alignment. A detailed evaluation of the morphology of the osteotomy surface with varying alignments is required. This could become an important indicator for defining an appropriate implant design for each target alignment.

## Conclusions

The posterolateral overhang of the lateral posterior condyle occurs when a symmetrical prosthesis is used. This overhang is expected to be improved by using an asymmetric implant with a small, rounded proximal portion of the lateral posterior condyle. The results of the present study show morphological information that is very impactful for TKA implant design. Further research is warranted to determine how the asymmetry of implant geometry affects knee biomechanics and kinematics and whether it truly has a positive clinical effect.
